# Personal

**Published:** 1894-06

**Authors:** 


### Personal.

Drs. C. E. Long and A. H. Macbeth, of Buffalo, have been appointed by the Health Commissioner, Dr. Wende, as sanitary inspectors of the Board of Health, and were assigned to duty early in May to inspect trains at Niagara Falls as a precaution against the importation of smallpox from Chicago. Dr. John A. Pettit, Deputy Health Commissioner, and Drs. F. A. Harrington and Edward L. Frost were assigned to a similar duty at the Buffalo railway stations, and Drs. Monroe Manges and Harry Mead performed a corresponding duty in relation to the incoming boats.

Dr. and Mrs. George H. Rohé, of Catonsville, Md., sailed for Europe, April 28, 1894, by the Etruria, to be absent about three months. Dr. Rohé went as the official delegate from the American Association of Obstetricians and Gynecologists to the Berlin Society of Obstetricians and Gynecologists at its fiftieth anniversary, celebrated May 9 and 10, 1894. Dr. Rohé will also investigate the construction and management of insane hospitals during his European tour.

Dr. J. B. Murphy, of Chicago, has been appointed honorary president for America of the surgical section of the twelfth International Medical Congress, to be held in St. Petersburg. The other appointments in this section were v. Bergmann, of Berlin, for Germany; Kocher, of Berne, for Switzerland; Sir William Stokes, of Dublin, for Ireland; Sir William MacCormac, of London, for England; Macewen, of Glasgow, for Scotland, and Mikulicz, of Vienna, for Austria.

Dr. Ira C. Brown, of Buffalo, is making a tour to California, having left Buffalo May 14th, to return about June 20, 1894, during which time he will attend the meeting of the American Medical Association in San Francisco.

---

Dr. H. Y. Grant announces the removal of his office from 50 West Tupper street to 414 Delaware, corner of Edward street, Buffalo. Hours, 9 A. M. to 12.30 P. M., 3.30 to 4.30 P. M.

---

Dr. N. G. Richmond, of Fredonia, attended the commencement exercises of Niagara University Medical School, including the Alumni dinner, in this city, May 9, 1894.

---

Dr. Andrew F. Currier has removed from 159 East 37th street to 138 Madison avenue, New York. Hours, 11 to 1. Residence, 104 Cottage avenue, Mt. Vernon.

---

Dr. William G. Taylor, clinical assistant in obstetrics at Niagara University, has removed from 66 West Chippewa street to 199 Franklin street, Buffalo.

---

Dr. Albert L. D. Campbell, of Mount Morris, N. Y., has been appointed health officer of that village for the ensuing year.

---

Dr. W. S. Tremaine has removed from 217 Franklin street to 410 Elmwood avenue. Hours, 10 to 12 A. M., 7 to 8 P. M.

---

Dr. John D. Flagg, of Buffalo, has removed from 93 West Mohawk street to 322 Pennsylvania street.

---

Dr. F. W. Hinkel, of Buffalo, has removed from 305 Delaware avenue to 274 Delaware avenue.

---

Dr. J. M. Hewitt, of Buffalo, has removed from 342 Swan street to 55 West Mohawk street.

---

Dr. A. M. Ewing, of Buffalo, has removed from 187 Delaware avenue to 143 Allen street.

Dr. J. J. Finerty, formerly of Erie, Pa., has removed to 179 Franklin street, Buffalo.

